# A kinematic convergence in ape and monkey vertical climbing informs debates on early hominin arborealism

**DOI:** 10.1073/pnas.2608183123

**Published:** 2026-08-17

**Authors:** Luke D. Fannin, Carmen Pape, W. Scott McGraw

**Affiliations:** ^a^ Department of Anthropology, Dartmouth College, Hanover, NH 03755; ^b^ School of Psychology and Neuroscience, University of St Andrews, St Andrews KY169JP, United Kingdom; ^c^ Department of Anthropology, The Ohio State University, Columbus, OH 43210

**Keywords:** midfoot break, vertical climbing, *Australopithecus*, CLCA

## Abstract

Recent debates on early hominin arborealism center around interpretations of fossilized foot bones as being either “African ape”- or “monkey”- like in their morphology. These dueling interpretations fuel competing locomotor reconstructions of the last common ancestor of chimpanzees and humans (LCA), including disagreements about the prevalence of vertical climbing behaviors. Yet it remains unclear if possessing an ape- or monkey-like foot impacts the safety and efficiency of climbing, in part because few kinematic data exist on cercopithecoid monkeys moving in their natural habitats. Here, we present detailed kinematic data on the feet of a prolific vertically-climbing cercopithecoid monkey (*Cercocebus atys*; the sooty mangabey) in the wild. We show that sooty mangabeys exhibit an extraordinary degree of midfoot dorsiflexion (~46°) during vertical climbs, a value nearly indistinguishable in magnitude to the ankle dorsiflexion of chimpanzees and slightly larger than the ankle dorsiflexion of climbing rainforest hunter-gatherers. This result suggests that, regardless of overall pedal morphology, the fitness challenges posed by vertical movements produced a kinematic convergence in primate foot biomechanics to improve climbing safety. Even if the LCA retained a more monkey-like foot compared to later hominins, our findings suggest that vertical climbing would still be a plausible component of its locomotor repertoire.

An enduring debate in paleoanthropology concerns the locomotion of the last common ancestor of humans and chimpanzees (LCA or CLCA). Morphometric analyses of fossilized foot bones in early hominins feature prominently in many evolutionary reconstructions (1), resulting in two competing models of LCA positional behavior. One model proposes that the LCA foot was decidedly African ape-like, with skeletal traits (e.g., cuboid and talus morphology) shaped for terrestrial quadrupedalism and tree climbing (2–5). The second model uses other skeletal evidence (e.g., relative cuboid length; Fig. 1*A*) to contend that the LCA had feet more like generalized cercopithecoid monkeys than any living ape, arguing instead for an ancestral positional behavior premised on above-branch, clambering quadrupedalism (6–8). Both models differ in the evolutionary importance afforded to vertical climbing. In the former, it is a crucial hominization factor for bridging movements between the arboreal and terrestrial milieus (5). In the latter, it is viewed as incompatible with the more monkey-like foot proposed for the LCA (8).

**Fig. 1.**
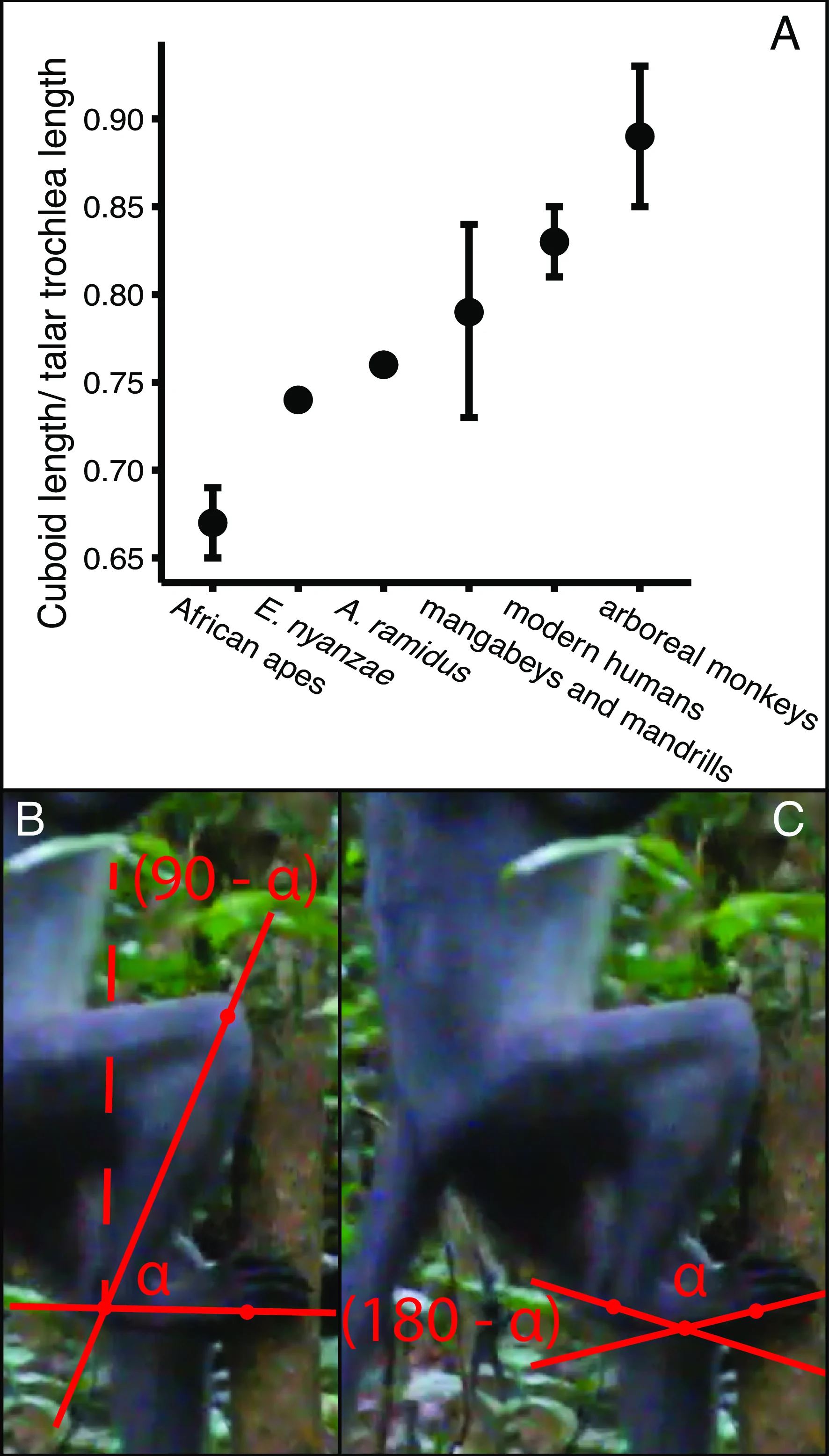
(*A*) Relative midfoot length across living and fossil primates. The early Miocene ape *Ekembo nyanzae* and hominin *Ardipithecus ramidus* have relative midfoot lengths that resemble terrestrial mangabeys and mandrills, a similarity used to argue for a monkey-like LCA foot (8). Black dots: mean; whiskers: 99% CI. (*B* and *C*) Vertical climbing behavior in a sooty mangabey (*Cercocebus atys*) in Taï Forest and the lower limb excursion angles measured: (*B*) *ankle dorsiflexion*; and (*C*) *midfoot dorsiflexion*. See *SI Appendix*, *Additional Methods* for angles used in analysis. α = measured angle.

The hypothesis that a monkey-like foot morphology would limit vertical climbing in the LCA has received some attention (5) but has yet to be properly field tested. In African apes, vertical climbing is facilitated by extreme dorsiflexion (>45°) at the tibiotalar (ankle) joint, which reduces the horizontal distance between the climber’s center of mass (COM) and the tree surface (9). This joint excursion moderates the downward pull of gravity by reducing potentially injurious backward pitching torques, thereby improving climbing safety (9). Accordingly, the anterior distal tibia (9) and anterior talar trochlea (5) of African apes are relatively more mediolaterally widened to facilitate routine extreme dorsiflexion. In contrast, most fossil hominins and modern humans possess a squarer and more symmetrical ankle joint relative to the African apes, making a similar degree of excursion challenging (9, 10). Instead of an expanded ankle joint, some modern populations—such as Twa hunter-gatherers—achieve comparable dorsiflexion by leveraging elongated gastrocnemius muscles (10). Cercopithecoid monkeys also lack a mediolaterally expanded anterior ankle joint (9); however, they may improve climbing performance through another means: a highly flexible midfoot, or midfoot “break” (9). Formed primarily at the lateral midfoot between the cuboid and the fourth and fifth metatarsals (11), monkey midfoot dorsiflexion during vertical climbing may be kinematically analogous to the extreme dorsiflexion of ape ankles (9). Increased midfoot mobility, in turn, may have provided an alternative means of improving vertical climbing safety in a “(LCA) midfoot resemble[ing] that of a generalized cercopithecoid more than that of a specialized African ape” (8: 4).

Here, we present systematic pedal kinematic data on vertical climbing in wild cercopithecoid monkeys, focusing on the midfoot and ankle joints (Fig. 1 *B* and *C*). Our study species, the sooty mangabey (*Cercocebus atys*), is a large, forest-dwelling African monkey that is a terrestrial forager and climber of vertical trunks (12). We present our findings here within a larger comparative analysis of primate foot kinematics, testing the hypothesis that an extreme degree of dorsiflexion—whether in the midfoot or ankle—is a critical shared adaptation for all primate vertical climbers (8, 9).

## Results

**Fig. 2.**
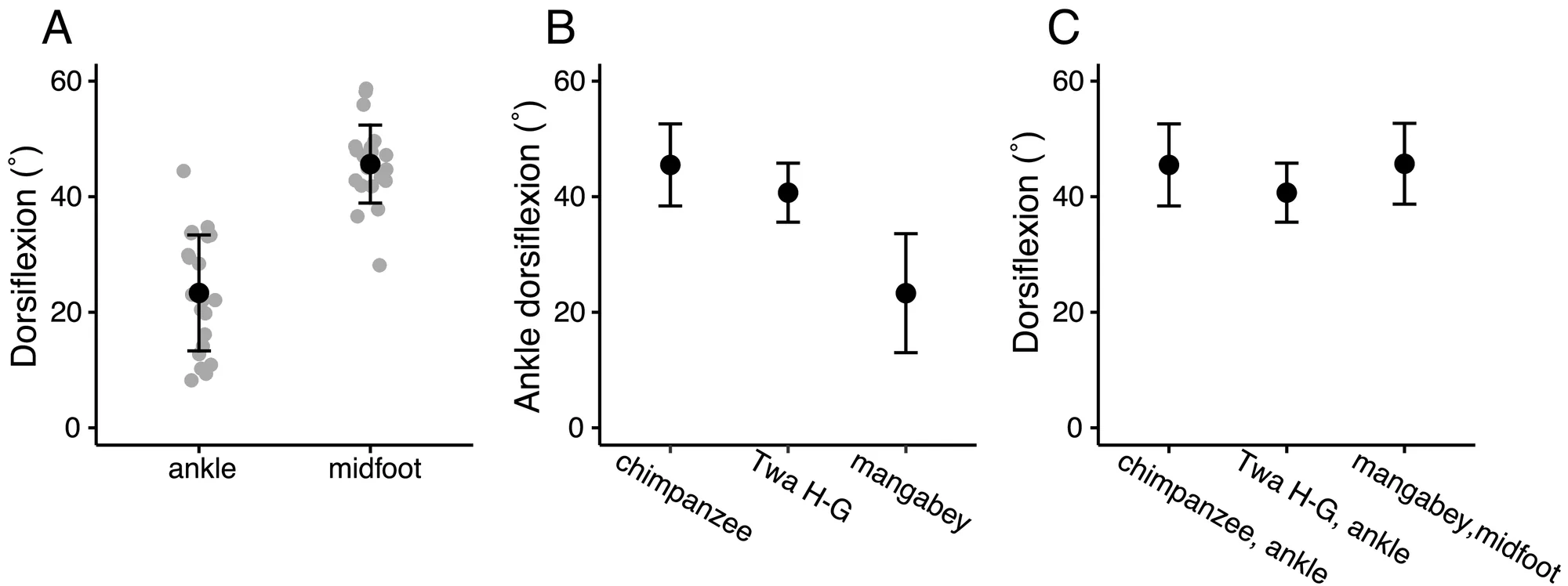
Comparative analysis of climbing kinematics. (*A*) Sooty mangabey midfoot dorsiflexion was significantly greater than ankle dorsiflexion during vertical climbing (within-subjects Wilcoxon test: S = 116, *P* < 0.0001). (*B*) Mangabey ankle dorsiflexion during vertical climbing was significantly lower than the ankle dorsiflexion of chimpanzees (Wilcoxon two-sample test: Z = −6.3, *P* < 0.0001) and Twa hunter-gatherers (Z = 3.6, *P* = 0.0004). (*C*) In contrast, average mangabey midfoot dorsiflexion during vertical climbing was statistically indistinguishable from both groups (chimpanzees: Z = −0.3, *P* = 0.75; Twa: Z = −1.8, *P* = 0.07). Black dots: mean; whiskers: ±1 SD.

Sooty mangabeys exhibited ~65% more maximum midfoot dorsiflexion [45.7° ± 7.0 (mean ± SD), n = 21] than maximum ankle dorsiflexion (23.3° ± 10.3, n = 21) during their vertical climbing bouts (Fig. 2*A*). Mangabey maximum ankle dorsiflexion was 65 and 54% less than the average maximum ankle dorsiflexion of chimpanzees (45.5° ± 7.1, n = 63) and climbing Twa hunter-gatherers (40.7° ± 5.1, n = 7), respectively (Fig. 2*B*). In contrast, mangabey maximum midfoot dorsiflexion was nearly identical to the maximum ankle dorsiflexion of chimpanzees and 12% higher than Twa maximum ankle dorsiflexion (Fig. 2*C*). Sooty mangabey maximum midfoot dorsiflexion was also larger than the maximum midfoot dorsiflexion previously recorded for chimpanzees during terrestrial quadrupedalism (26.5° ± 4.4, n = 10) and for walking modern humans (11.1° ± 2.4, n= 32). During maximum climbing dorsiflexion, chimpanzees and sooty mangabeys were comparable in the average size-standardized horizontal distance between their COM and the tree (*SI Appendix*, *Additional Methods*).

## Discussion

We observed the vertical climbing of wild sooty mangabeys and recorded an exceptional degree of dorsiflexion at the midfoot. For context, the mean angle of dorsiflexion (≈46°) at the midfoot is comparable to the hyperdorsiflexion of chimpanzee ankles during vertical climbing. The broader significance of our findings is twofold. First, our findings suggest that extreme midfoot dorsiflexion is an alternative means of improving vertical climbing safety in primates that lack the anatomical capabilities for extreme ankle dorsiflexion (9). Second, our data suggest that if the LCA possessed a more monkey-like midfoot (8), vertical climbing would still be a plausible component of its locomotor repertoire, along with the previously hypothesized above-branch clambering behaviors (6–8). However, it is important to stress that our results do not favor a particular LCA morphological reconstruction. Rather, our comparative data illustrate a striking convergence in foot kinematics that improves climbing performance, suggesting that safe vertical climbing is readily accomplished and compatible with either an ape- or monkey-like foot.

Our findings also cast light on the functional significance of midfoot variability in early hominins. The ankles of most Plio-Pleistocene australopiths lacked ape-like mediolateral expansion of their anterior distal tibiae (9), suggesting they used minimal chimpanzee-like flexed ankle vertical climbing and more frequent striding bipedalism (1). Yet several *Australopithecus* species also possessed unique fourth metatarsal (Mt4) morphologies—such as the relatively short dorsoplantar height of the Mt4 base in *A. deyiremeda* (~3.5 Ma) and the strongly convex Mt4 base in *A. sediba* (~2.0 Ma)—that suggest a greater degree of midfoot flexibility compared to modern humans (13, 14 but see ref. 1). Previous authors proposed that the greater midfoot mobility of these taxa could be “useful for climbing” (15: 646) or indicative of a hominin that “had re-evolved a more arboreal lifestyle from a more terrestrial ancestor” (14: 4). Our kinematic analysis supports these functional interpretations and hints that a cercopithecoid-like midfoot dorsiflexion—perhaps similar in magnitude to the mangabeys here—may have offered these hominins an alternative means for safe vertical climbing (9).

## Materials and Methods

We opportunistically filmed habituated wild sooty mangabeys in the primary study grid of the Taï Monkey Project in Taï National Park, Côte d’Ivoire at 30 fps using a tripod-mounted Canon EOS 7D video camera. Our video field collection protocol followed (9) and our analytical protocol for calculating maximum ankle and midfoot dorsiflexion angles followed (9–11), respectively (*SI Appendix*, *Additional Methods*).

## Supplementary Material

Appendix 01 (PDF)

Dataset S01 (XLSX)

Dataset S02 (XLS)

Dataset S03 (XLSX)

Dataset S04 (PDF)

## Data Availability

Study data are included in the article and/or supporting information.

## References

[1] J. DeSilva, E. McNutt, J. Benoit, B. Zipfel, One small step: A review of Plio-Pleistocene hominin foot evolution. Am. J. Phys. Anthropol. **168**, 63–140 (2019). 30575015 10.1002/ajpa.23750

[2] D. L. Gebo, Climbing, brachiation, and terrestrial quadrupedalism: Historical precursors of hominid bipedalism. Am. J. Phys. Anthropol. **101**, 55–92 (1996). 8876814 10.1002/(SICI)1096-8644(199609)101:1<55::AID-AJPA5>3.0.CO;2-C

[3] T. C. Prang, The African ape-like foot of *Ardipithecus ramidus* and its implications for the origin of bipedalism. eLife **8**, e44433 (2019). 31038121 10.7554/eLife.44433 PMC6491036

[4] T. C. Prang, New analyses of the *Ardipithecus ramidus* foot provide additional evidence of its African ape–like affinities: A reply to Chaney et al. (2021). J. Hum. Evol. **164**, 103135 (2022). 35164965 10.1016/j.jhevol.2021.103135

[5] T. C. Prang, M. W. Tocheri, B. A. Patel, S. A. Williams, C. M. Orr, *Ardipithecus ramidus* ankle provides evidence for African ape-like vertical climbing in the earliest hominins. Commun. Biol. **8**, 1454 (2025). 41094103 10.1038/s42003-025-08711-7 PMC12528372

[6] C. O. Lovejoy, B. Latimer, G. Suwa, B. Asfaw, T. D. White, Combining prehension and propulsion: The foot of *Ardipithecus ramidus*. Science **326**, 72e1–8 (2009). 19810198

[7] C. O. Lovejoy, G. Suwa, S. W. Simpson, J. H. Matternes, T. D. White, The great divides: *Ardipithecus ramidus* reveals the postcrania of our last common ancestors with African apes. Science **326**, 73–106 (2009). 19810199

[8] M. E. Chaney, C. A. Ruiz, R. S. Meindl, C. O. Lovejoy, The foot of the human–chimpanzee last common ancestor was not African ape-like: A response to Prang (2019). J. Hum. Evol. **164**, 102940 (2022). 33441261 10.1016/j.jhevol.2020.102940

[9] J. M. DeSilva, Functional morphology of the ankle and the likelihood of climbing in early hominins. Proc. Natl. Acad. Sci. U.S.A. **106**, 6567–6572 (2009). 19365068 10.1073/pnas.0900270106 PMC2672491

[10] V. V. Venkataraman, T. S. Kraft, N. J. Dominy, Tree climbing and human evolution. Proc. Natl. Acad. Sci. U.S.A. **110**, 1237–1242 (2013). 23277565 10.1073/pnas.1208717110 PMC3557098

[11] J. M. DeSilva, Revisiting the “midtarsal break”. Am. J. Phys. Anthropol. **141**, 245–258 (2010). 19672845 10.1002/ajpa.21140

[12] L. D. Fannin, M. S. Joy, N. J. Dominy, W. S. McGraw, J. M. DeSilva, Downclimbing and the evolution of ape forelimb morphologies. R. Soc. Open Sci. **10**, 230145 (2023). 37680499 10.1098/rsos.230145 PMC10480693

[13] Y. Haile-Selassie , A new hominin foot from Ethiopia shows multiple Pliocene bipedal adaptations. Nature **483**, 565–569 (2012). 22460901 10.1038/nature10922

[14] J. M. DeSilva , The lower limb and mechanics of walking in *Australopithecus sediba*. Science **340**, 1232999 (2013). 23580534 10.1126/science.1232999

[15] Y. Haile-Selassie , New finds shed light on diet and locomotion in *Australopithecus deyiremeda*. Nature **648**, 640–648 (2025). 41299167 10.1038/s41586-025-09714-4 PMC12711576

