# Supplementary material for: A kinematic convergence in ape and monkey vertical climbing informs debates on early hominin arborealism
Source: Proc Natl Acad Sci U S A. 2026 Aug 17;123(35):e2608183123. doi: 10.1073/pnas.2608183123 (PMC13535176; doi:10.1073/pnas.2608183123)
Supplement: Supplementary file 1 [file pnas.2608183123.sapp.pdf]

PNAS

Supporting Information for

A kinematic convergence in ape and monkey vertical climbing informs debates on early hominin arborealism.

Luke D. Fannin[1,2]
Carmen Pape[3]
W. Scott McGraw[3]

(1) *Department of Anthropology, Dartmouth College, Hanover, NH, USA*
(2) *School of Psychology and Neuroscience, University of St Andrews, St Andrews, UK*
(3) *Department of Anthropology, The Ohio State University, Columbus, OH, USA*

Luke D. Fannin
Email: luke.daniel.fannin@dartmouth.edu

This PDF file includes:

Supporting Information Text (Additional Methods)
Legends for Datasets S1-S3
SI References

Other supporting materials for this manuscript include the following:

Dataset S1
Dataset S2
Dataset S3

Supporting Information Text

Additional Methods

Video capture and kinematic analyses. We observed sooty mangabeys (*Cercocebus atys*) in the primary study grid of the Taï Monkey Project in Taï National Park, Côte d'Ivoire in August-September 2019 and January-March 2022. We targeted adults and subadults, although some older juveniles were also recorded opportunistically (*Dataset S1*). Our filming distances ranged between 5 and 10m, but every vertical climbing bout was recorded in lateral view and limited to the first 2-5m to minimize angle induced errors (1). We isolated video stills that depicted the near-camera foot in the "support" phase of climbing (2,3) and then utilized the angle tool in ImageJ/Fiji (4,5) to measure joint excursion angles. We selected video stills depicting the maximum dorsiflexion angle of each climbing bout in parasagittal view. We then measured these maximum talocrural and midfoot dorsiflexion angles in the same video frame. Of the approximately 180 videos taken for this study, 21 (12%) provided a completely unobscured view of the near ankle joint at the appropriate viewing height. Stills from these videos were subsequently measured for ankle and midfoot dorsiflexion (*Figure 1B,C*).

We estimated maximum ankle dorsiflexion following DeSilva (2) and Venkataraman et al. (3) as the angle between two lines: the first line bisected the tibia, running from the knee joint to the heel; the second line ran from the heel to the metatarsophalangeal joint of the fifth metatarsal. After measuring this angle using the Image J angle tool (represented in *Figure 1b* as α), this angle was then subtracted from 90° (90-α), to match previously published ankle kinematic data from chimpanzees and Twa hunter-gathers. We estimated maximum midfoot dorsiflexion following DeSilva (6) and DeSilva and Gill (7): the first line was drawn between the heel and the tuberosity of the fifth metatarsal (e.g., the approximate location of the cuboid-metatarsal joint); the second line was drawn along the plantar part of the forefoot, between the cuboid-metatarsal joint and the metatarsophalangeal joint of the fifth metatarsal (*Figure 1c*). This angle was then subtracted from 180 (180-α) —also in-line with previous studies of primate midfoot kinematics (6,7)— so that as midfoot dorsiflexion increased, the excursion angle becomes larger. This adjustment made our midfoot kinematic measurements more comparable to those estimated for ankle dorsiflexion. Although there is likely additional midfoot mobility present at the calcaneocuboid joint, we chose to focus on the cuboid-metatarsal joint because it is the site of maximum midfoot dorsiflexion for primates in both terrestrial and arboreal contexts (6).

We used JMP 18 (SAS) for all our analyses. We carried out our within- and between-sample comparisons using two-tailed non-parametric Wilcoxon signed rank tests. We were able to identify age-sex classes in all 21 mangabey climbing videos, but the difficulty of field filming conditions made getting some individual IDs during filming challenging, an important study limitation. Because many group females are difficult to identify in video footage, some individuals may have been re-sampled, which may violate the assumed statistical independence among data points and necessitated our use of non-parametric statistics. Still, our non-parametric results mirrored those from two-tailed parametric t-tests: (mangabey midfoot vs. ankle: within-subjects t test, $t(20) = 9.64$; $p < 0.0001$; chimpanzee ankle vs. mangabey ankle: Welch's t, $t(26.6) = -9.2$, $p < 0.0001$; Twa hunter-gatherer ankle vs. mangabey ankle: $t\ (21.3) = -5.9$, $p < 0.0001$; chimpanzee ankle vs. mangabey midfoot: $t\ (34.7) = 0.1$, $p = 0.95$; Twa hunter-gatherer ankle vs. mangabey midfoot: $t(14.1) = 2.0$, $p = 0.07$), suggesting that our comparisons are recovering a biologically-meaningful pattern regardless of our choice of statistical test.

Intra- and inter-observer error. For our evaluation of intra-observer error, LDF coded a subset of climbing videos (n = 11) again for ankle and midfoot dorsiflexion angles. The average measurement error for ankle dorsiflexion and midfoot dorsiflexion across the ten videos was +/- 3.5% and +/6.4%, respectively. These error values compare favorably to previous kinematic studies of wild primate ankles (2). Additionally, the average ankle and midfoot dorsiflexion angles calculated in the intra-observer analysis were not significantly different from the average values of the same 11 videos

from the original dataset (ankle: 28.3° vs. 27.2°, t (19.3) = -0.27, p = 0.78; midfoot: 46.6° vs. 46.5°, t (19.9) = -0.04, p = 0.97), suggesting that our reported sample means are generally robust to intra-observer variation in landmark placement.

For our evaluation of inter-observer error, CP coded a larger subset of climbing videos (n=16), and her values were compared to the original angles collected by LDF. The average measurement error for ankle and midfoot dorsiflexion across the 16 videos was +/- 5.0% and 6.5%, respectively. These error values also compare favorably to previous kinematic studies of wild primate ankles (2). Additionally, the average ankle and midfoot dorsiflexion angles calculated in the inter-observer analysis were not significantly different from the average values of the same 16 videos from the original dataset (ankle: 25.3° vs. 20.3°, t (29.6) = -1.32, p = 0.20; midfoot: 44.8° vs. 45.1°, t(28.6) = 0.11, p = 0.91), further suggesting that our reported sample means remain robust to inter-observer variation in landmark placement.

COM analysis of vertical climbs. In a subset of chimpanzee and mangabey video stills (N=10 per group), we estimated the relative distance between the climber center of mass (COM) and the surface of the tree trunk closest to the COM. We used this as a proxy for measuring climbing performance between the two primate groups, as similar degrees of dorsiflexion should result in a similar degree of reduction in the moment arm of the destabilizing gravitational pitching torque once the effects of body size are controlled. In Image J, we measured the pixel distance between the center of each animal's thorax and the closest parallel point on the tree's trunk (referencing the climbing free body diagrams in previous publications to estimate the approximate location of the COM (8–11)). We then scaled this value to the pixel length of the hindlimb nearest the camera view, calculating hindlimb length as the sum of: (1) straight line bisecting the upper leg between hip joint and the knee joint; and (2) a straight line bisecting the lower leg between the knee and ankle joints. A larger ratio indicates a relatively greater distance between the COM and the tree (scaled by respective species' leg length), suggesting a greater pitching torque.

Mangabeys possessed a relatively smaller leg-length-scaled horizontal distance between the tree and their COM, on average, than chimpanzees (mangabeys: 0.48 ± 0.09; chimpanzees: 0.53 ± 0.07), although these differences were not statistically significant when compared parametrically (Welch's t (16.3) = 1.62, p = 0.12) or non-parametrically (Wilcoxon two-sample test: Z = 1.1, p = 0.27)

Additional comparative kinematic data. We collected previous kinematic measurements of maximum ankle dorsiflexion in vertically-climbing chimpanzees and modern human populations of hunter gathers (consenting Twa men, all of which were experienced honey-gathers) from DeSilva (2) and Venkataraman et al., (3), respectively. Midfoot dorsiflexion data on chimpanzees and modern humans were sourced from DeSilva (6) and DeSilva and Gill (7), respectively. Chimpanzees engage in some midfoot dorsiflexion during vertical climbs (2), but only during the subsequent push off phase of climbing, after maximum dorsiflexion of the ankle is achieved. From the perspective of minimizing gravitational torque during the support phase of climbing, the most functionally-and kinematically-analogous comparison is between ape ankle dorsiflexion and monkey midfoot dorsiflexion, as argued previously (2,10). Chimpanzee average maximum midfoot dorsiflexion in DeSilva (6) was recorded on captive chimpanzees walking quadrupedally on the ground. Although we did not collect estimates of chimpanzee midfoot dorsiflexion during vertical climbs, experimental work by Holowka et al. (12) found that peak midfoot dorsiflexion kinematics were similar between terrestrial quadrupedalism and vertical climbing in captive chimpanzees (an average difference of only 2°; their Table 5). We therefore felt comfortable making this comparison in our results but acknowledge that further data on wild chimpanzee midfoot kinematics during vertical climbing is a priority for future work.

Comparative fossil data from Figure 1. All cuboid and talar measurements reported in Figure 1a were taken from Prang (13), except those of *Ekembo nyanzae*, which were taken on high quality casts of the original fossils in the Dartmouth Paleoanthropology Lab following similar measurement protocols. We report relative cuboid length as the ratio of cuboid length to talar trochlear length

(14). The ratio reported for *E. nyanzae* is a composite measurement from two individuals, composed of the talar fossil KNM-RU 1743 and cuboid fossil KNM-RU 5872. These fossils have been combined in previous analyses (e.g., Walker and Pickford (15)). The measurements of the *Ardipithecus ramidus foo*t, by contrast, were taken by Prang (13) from the same individual (ARA-VP 6/500). Our modern morphological sample from Prang (13) comprised African great apes (N= 115), mangabeys and mandrills (N = 14), modern humans (N = 52); and arboreal African cercopithecoids (N = 29). We classified the arboreal mangabeys (*Lophocebus*) in the arboreal cercopithecoid group because, unlike *Cercocebus* and *Mandrillus*, they are primarily arboreally-adapted and rarely vertical climb (16,17).

Ethics statement. Our field collection protocol followed ASP principles for the ethical treatment of non-human primates, and it was approved by IACUC, Ohio State University (protocol 2008A0051-R4).

Dataset S1 (separate file). Mangabey midfoot and ankle kinematic data collected in the current study and other comparative ankle kinematic data. Measurement terminology at headers follows Figure 1.

Dataset S2 (separate file). Hindlimb-scaled horizontal differences between the COM and the tree surface in chimpanzees and mangabeys.

Dataset S3 (separate file). Relative cuboid length measurements from the living and fossil primates in Figure 1A.

SI References

1. L. D. Fannin, M. S. Joy, N. J. Dominy, W. S. McGraw, J. M. DeSilva, Downclimbing and the evolution of ape forelimb morphologies. *R. Soc. Open Sci.* 10, 230145 (2023).
2. J. M. DeSilva, Functional morphology of the ankle and the likelihood of climbing in early hominins. *Proc. Natl. Acad. Sci. USA* 106, 6567–6572 (2009).
3. V. V. Venkataraman, T. S. Kraft, N. J. Dominy, Tree climbing and human evolution. *Proc. Natl. Acad. Sci. USA* 110, 1237–1242 (2013).
4. C. A. Schneider, W. S. Rasband, & K. W. Eliceiri, NIH Image to ImageJ: 25 years of image analysis. *Nat. Methods* 9, 671–675 (2012).
5. J. Schindelin, *et al.*, (2012). Fiji: an open-source platform for biological-image analysis. *Nat. Methods* 9, 676–682 (2012).
6. J. M. DeSilva, Revisiting the "midtarsal break". *Am. J. Phys. Anthropol.* 141, 245–258 (2010).
7. J. M. DeSilva, S. V. Gill, Brief communication: a midtarsal (midfoot) break in the human foot. *Am. J. Phys. Anthropol.* 151, 495–499 (2013).
8. M. Cartmill, "Pads and claws in arboreal locomotion" in *Primate Locomotion*., F. A. Jenkins, Ed. (Academic Press, 1974), pp. 45–83.
9. M. Cartmill, "Climbing" in *Functional Vertebrate Morphology*., M. Hildebrand, D. M. Bramble, K. F. Liem, D. B. Wake, Eds. (Belknap Press, 1985), pp. 73–88.
10. J. M. DeSilva, "Vertical climbing adaptations in the anthropoid ankle and midfoot: implications for locomotion in Miocene catarrhines and Plio-Pleistocene hominins," University of Michigan, Ann Arbor, MI (2008).
11. T. C. Prang, M. W. Tocheri, B. A. Patel, S. A. Williams, C. M. Orr, *Ardipithecus ramidus* ankle provides evidence for African ape-like vertical climbing in the earliest hominins. *Commun. Biol.* 8, 1454 (2025).
12. N. B. Holowka, M. C. O'Neill, N. E. Thompson, B. Demes, Chimpanzee ankle and foot joint kinematics: Arboreal versus terrestrial locomotion. *Am. J. Phys. Anthropol.* 164, 131–147 (2017).
13. T. C. Prang, The African ape-like foot of *Ardipithecus ramidus* and its implications for the origin of bipedalism. *eLife* 8, e44433 (2019).

14. M. E. Chaney, C. A. Ruiz, R. S. Meindl, C. O. Lovejoy, The foot of the human–chimpanzee last common ancestor was not African ape-like: A response to Prang (2019). *J. Hum. Evol.* 164, 102940 (2022).
15. A. C. Walker, M. Pickford, "New postcranial fossils of *Proconsul africanus* and *Proconsul nyanze*", in *New Interpretations of Ape and Human Ancestry*., R. L. Ciochon, R. S. Corruccini, Eds. (Plenum Press, 1983), pp. 325–351.
16. J. G. Fleagle, W. S. McGraw, Skeletal and dental morphology supports diphyletic origin of baboons and mandrills. *Proc. Natl. Acad. Sci USA* 96, 1157–1161 (1999).
17. J. G. Fleagle, W. S. McGraw, Skeletal and dental morphology of African papionins: unmasking a cryptic clade. *J. Hum. Evol.* 42, 267–292 (2002).
