# Supplementary material for: A kinematic convergence in ape and monkey vertical climbing informs debates on early hominin arborealism
Source: Proc Natl Acad Sci U S A. 2026 Aug 17;123(35):e2608183123. doi: 10.1073/pnas.2608183123 (PMC13535176; doi:10.1073/pnas.2608183123)
Supplement: Supplementary file 2 [file pnas.2608183123.sd04.pdf]

#Data read me file for Fannin et al., 2026
#Proceedings of the National Academy of Sciences

#Datasets

Dataset S1 - Mangabey climbing kinematic data and comparative kinematic data

Page 1 - kinematic data for wild sooty mangabeys
-climbing.video.number - sample number for the video
-species - species of primate
-age sex class - estimated age sex class of individual from video
180-alpha - maximum midfoot dorsiflexion angle recorded from the video still
90-alpha - maximum ankle dorsiflexion recorded from the video still

Page 2 - kinematic data for wild Pan troglodytes (from DeSilva, 2009, PNAS)
Date - date video was collected
Time - time video was collected
Chimpanzee - individual ID, if known
Angle of Dorsiflexion - angle of dorsiflexion of ankle
Source- source of kinematic data

Page 3- kinematic data for Twa hunter-gatherers (From Venkataraman et al., 2013, PNAS).
Generic ID - ID of individual trial
Subject ID - ID of climbing individual (anonymous)
Angle of Dorsiflexion - angle of dorsiflexion of ankle

###
Dataset S2 - COM analysis of mangabeys and chimpanzees
Climb No - climbing frame studied
Species - species of primate
COM - length of COM of the thorax to parallel surface of the tree trunk in pixels
Hip to knee - length of the hip to knee joint in pixels
Knee to ankle - length of the knee to ankle joint in pixels
Total limb length - total length of the lower limb (hip to ankle joint) in pixels
COM to limb length ratio - ratio of the distance between the COM (thorax) and the parallel tree surface to the total hindlimb length

##
Dataset S3 - morphological data from living and fossil primates (from Prang, 2019 and collected from casts in the Dartmouth Paleoanthropology Lab)

Accession - accession number of the specimen
Taxon - taxon of the specimen
Category - category of classification used in figure 1a
Relative cuboid length - relative length of the cuboid, represented as the ratio of the

cuboid length divided by the length of the talar trochlea (measurements as in Prang, 2019, eLife).
